# Herniated Gallbladder Following a Bull Run: A Case Report

**DOI:** 10.7759/cureus.74354

**Published:** 2024-11-24

**Authors:** Ana Cláudia Soares, Ana Nunes Vieira, Inês Bagnari, Joana Bonança, Sara Leonor

**Affiliations:** 1 Surgery, Hospital de Santo Espírito da Ilha Terceira, Angra do Heroísmo, PRT

**Keywords:** blunt liver trauma, cholecystectomy laparoscopic, elective surgical procedures, gallbladder herniation, thoracoabdominal trauma

## Abstract

The liver is the most common organ injury associated with blunt trauma. Blunt hepatic trauma, due to the high kinetic impact on the liver, causes compression and parenchymal disruption, leading to tears in its vascular structures. By contrast, gallbladder injury is rare because it is located beneath and shielded by the liver. This report concerns a case where hepatic and abdominal wall tearing following blunt trauma led to gallbladder herniation.

## Introduction

Gallbladder herniation is an uncommon occurrence, with few case reports due to its rarity. Reported cases include gallbladder herniation through a ventral hernia [1], incarcerated inguinal hernia [2], incisional hernia [3], and even parastomal hernia [4]. To our knowledge, there are no documented cases of gallbladder herniation following blunt trauma.

This report describes a case of a herniated gallbladder after blunt trauma. Gallbladder injury is rare due to its position, covered by the liver, whereas the liver is the most commonly injured organ in blunt trauma. Although most hepatic injuries are minor and treated non-operatively, surgical intervention is required in 14% of patients [5] with blunt abdominal trauma. This report concerns a case where hepatic and thoracoabdominal wall tearing after blunt trauma led to gallbladder herniation. This case report was previously presented as a meeting poster at the 2024 European Congress of Trauma and Emergency Surgery on April 28th, 2024.

## Case presentation

We present a case of a healthy 60-year-old man with no prior health issues or surgical history, and no regular medications. The patient participated in a street rope bull run in Terceira Island, Azores, and was knocked down by a bull. This is a traditional event held regularly during the festive season.

On initial evaluation in the emergency department, he was hemodynamically stable and eupneic. On physical examination, he had pain and an abrasion in the anterior wall of the right lower hemithorax. A chest x-ray revealed fractures of the fourth to ninth right ribs on the right side. Blood workup showed a hemoglobin of 15.7g/dL (hematocrit: 44.6%), alanine transaminase (ALT) of 326 U/L, aspartate transaminase (AST) of 303 U/L, gamma-glutamyltransferase (GGT) of 96 U/L. Abdominal ultrasonography was unremarkable, showing no free fluid.

**Figure 1:**
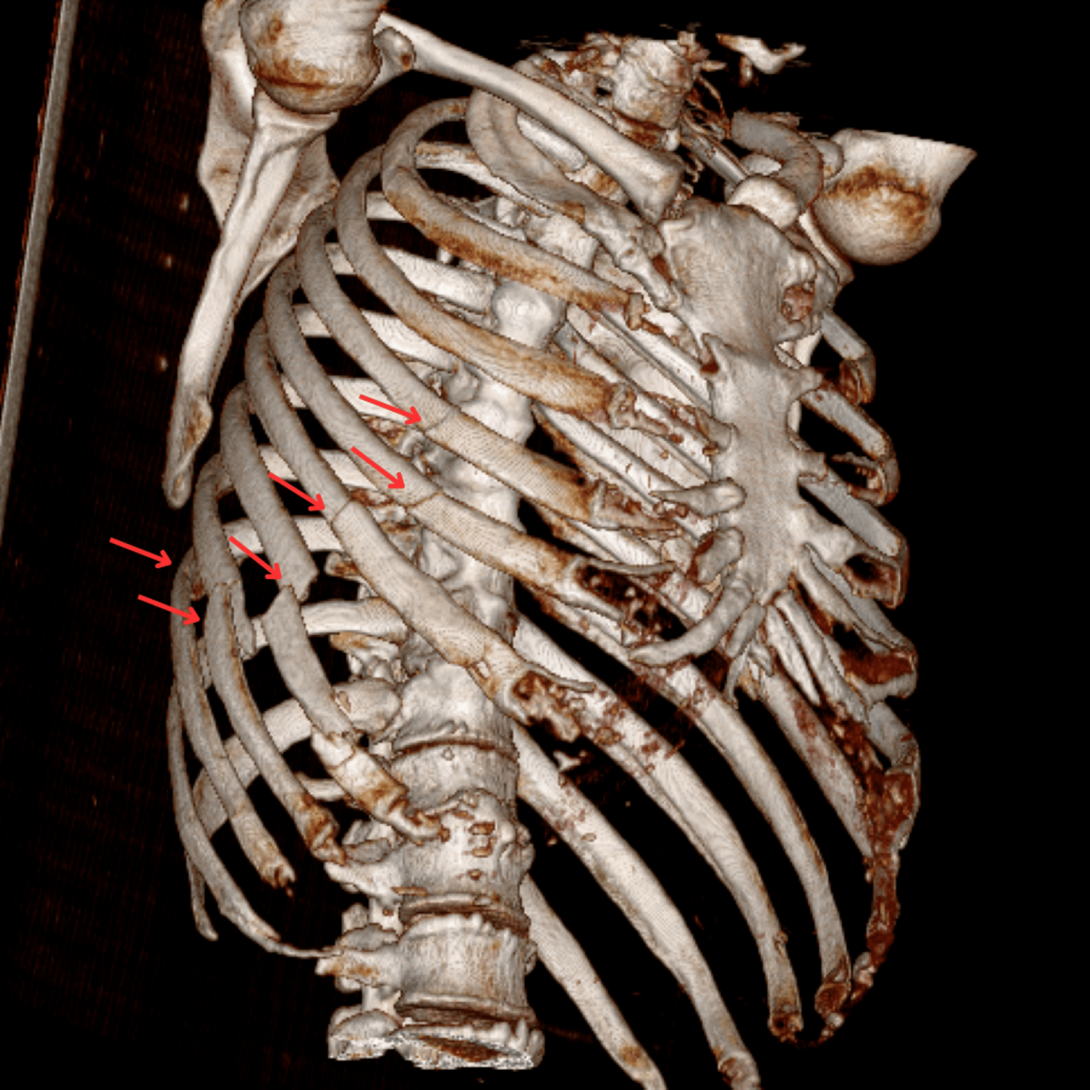
3D reconstruction on CT scan: fracture of 4th to 9th ribs (arrow).

**Figure 2:**
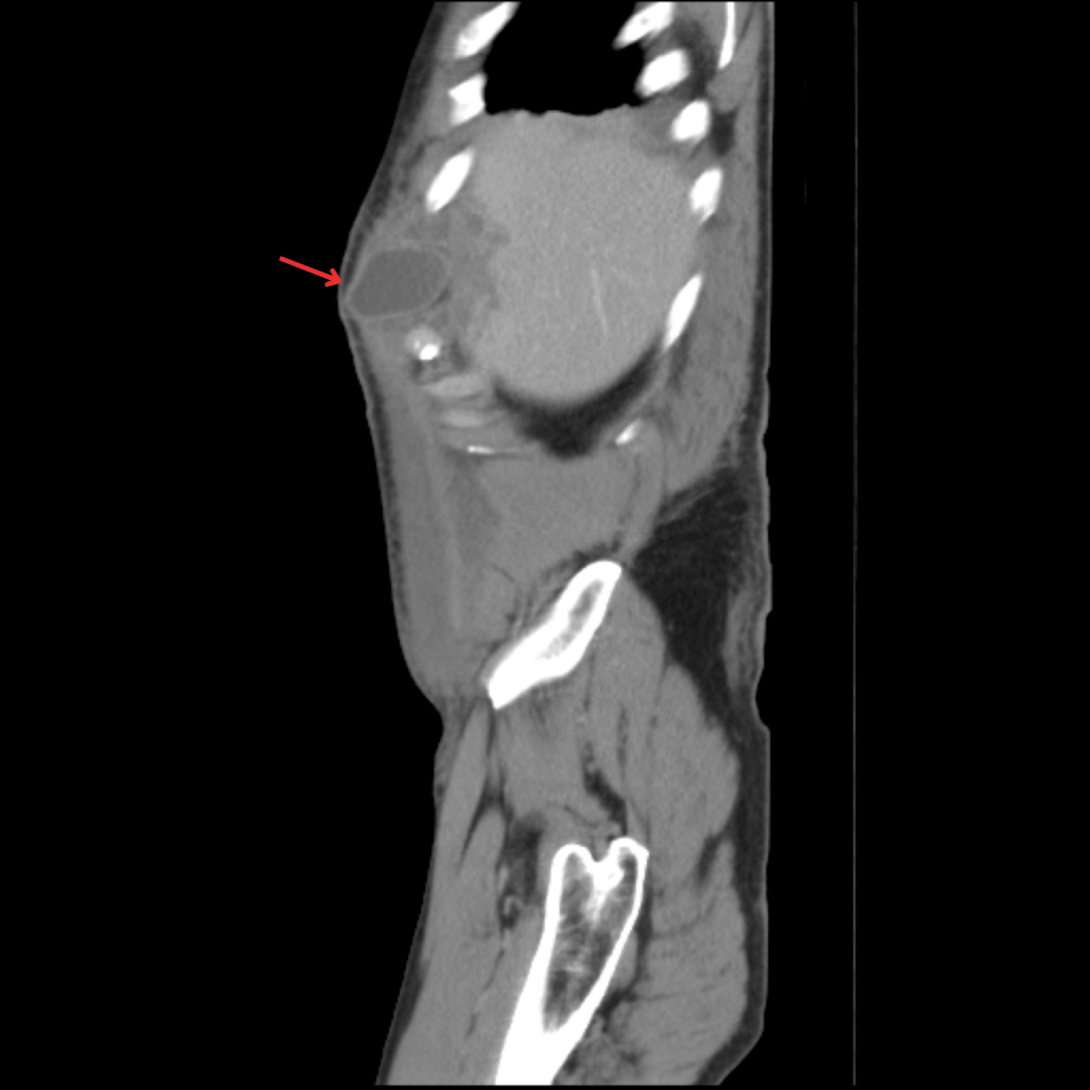
Herniated gallbladder (arrow), projected through the thoracoabdominal wall tearing.

**Figure 3:**
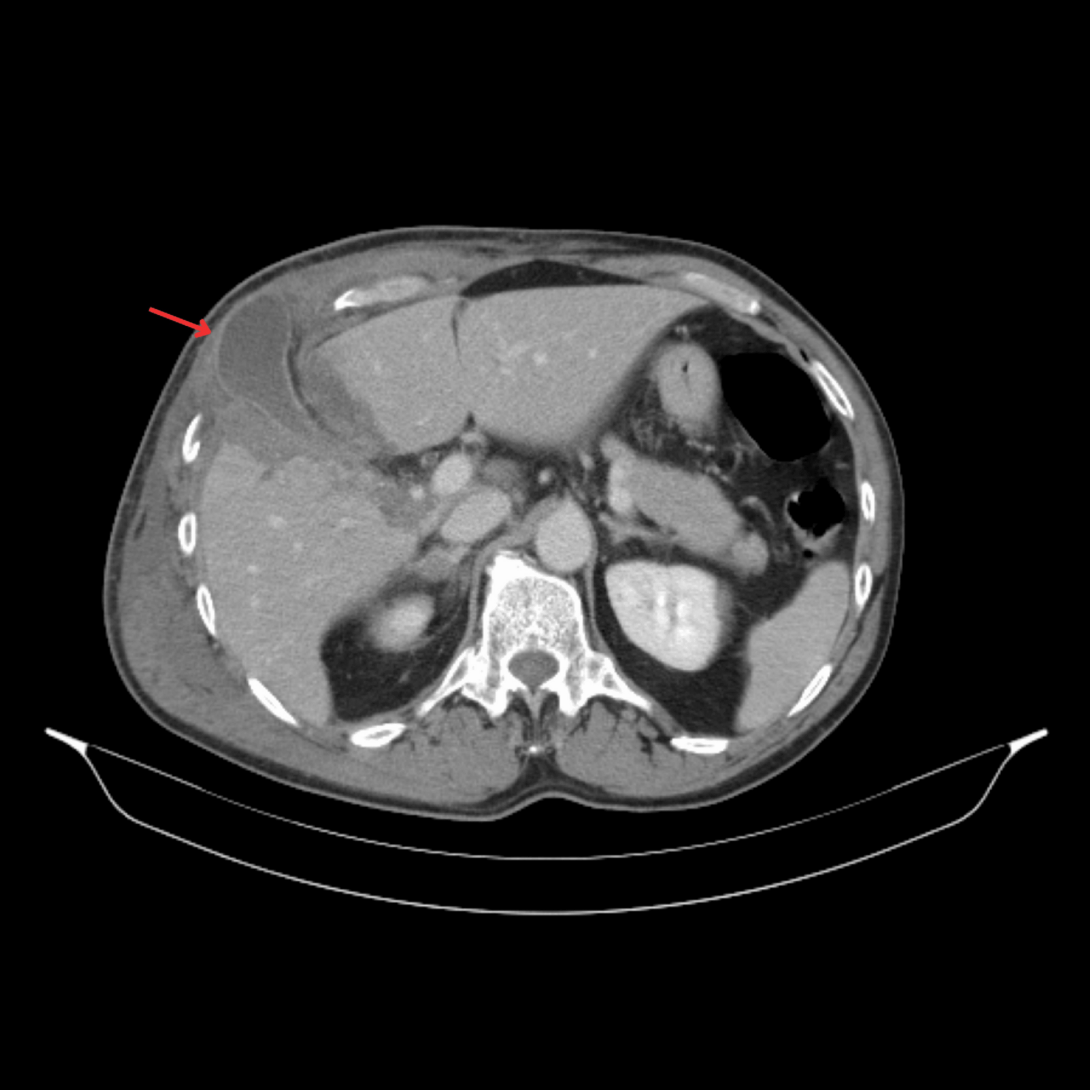
Hepatic laceration of the right lobe (VIII and IV segments) and herniated gallbladder (arrow) through the laceration.

**Figure 4:**
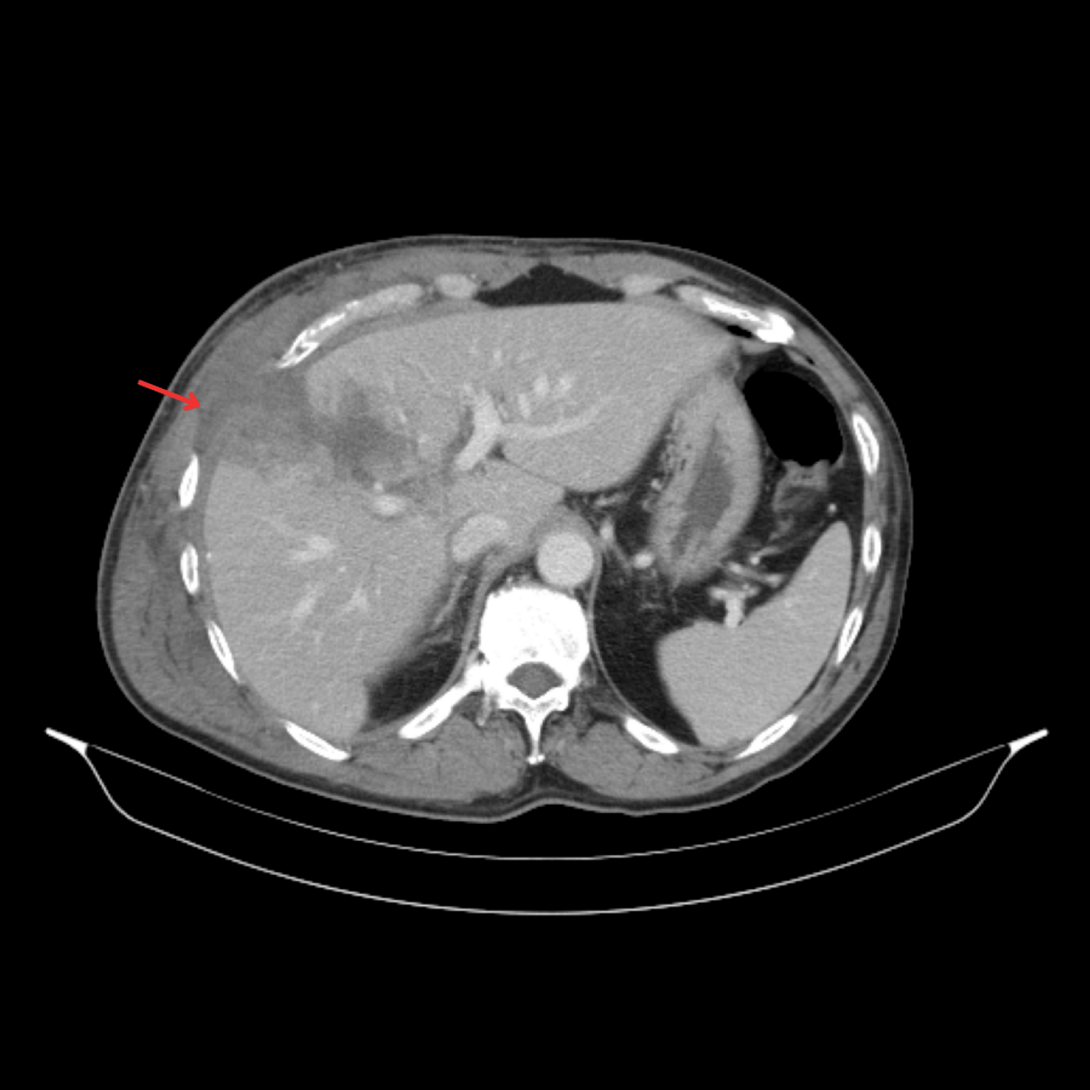
Hepatic laceration of the right lobe (VIII and IV segments) (arrow).

The patient was admitted for monitoring and pain management. A control blood workup, performed 48 hours after admission, revealed worsening liver function tests (ALT: 663 U/L, AST: 587 U/L, GGT: 221 U/L). A subsequent CT scan showed a grade III hepatic laceration [6] and disruption of the abdominal wall muscles. Alongside an associated rib fracture (Figure 1), these injuries led to the protrusion of the gallbladder (Figure 2), which was found lying in the subcutaneous tissue between the fractured sixth and seventh ribs (Figures 3, 4).

The case was discussed in a reference center for hepatobiliary surgery and liver transplantation, and conservative management was decided. Serial blood workup was performed and the lowest recorded hemoglobin level was 9.9 g/dL (hematocrit: 28.6%), four days after admission. The patient did not receive blood transfusions during the hospital stay. A control CT was performed nine days after the admission and it did not show escalation of the previous findings. The patient remained stable and was discharged after two weeks.

**Figure 5:**
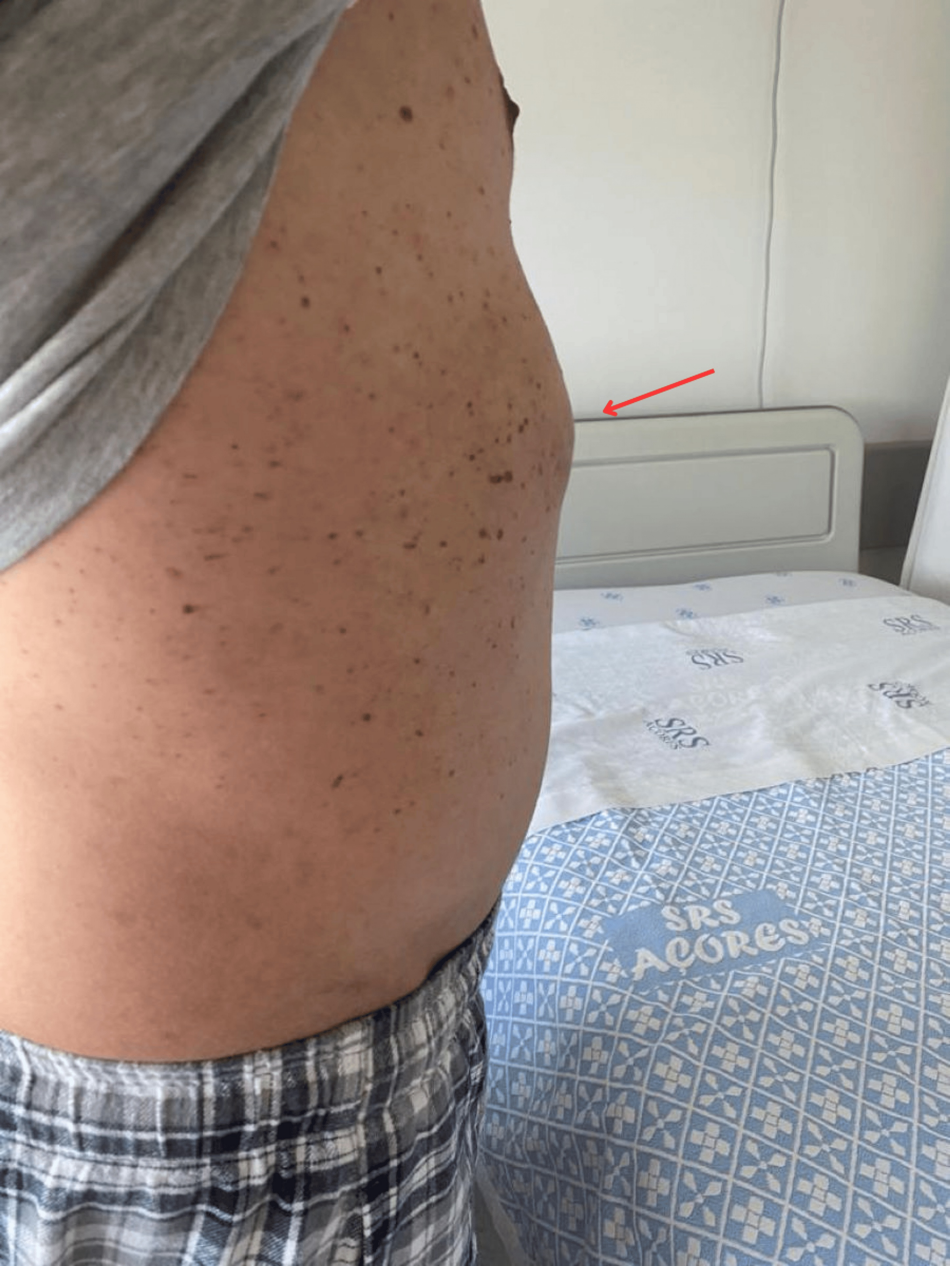
Bulging gallbladder (arrow) in the lower thoracic wall.

In the following months the patient was regularly reevaluated, and nine months after the traumatic event, elective surgery was performed (Figure 5).

Surgery description

**Figure 6:**
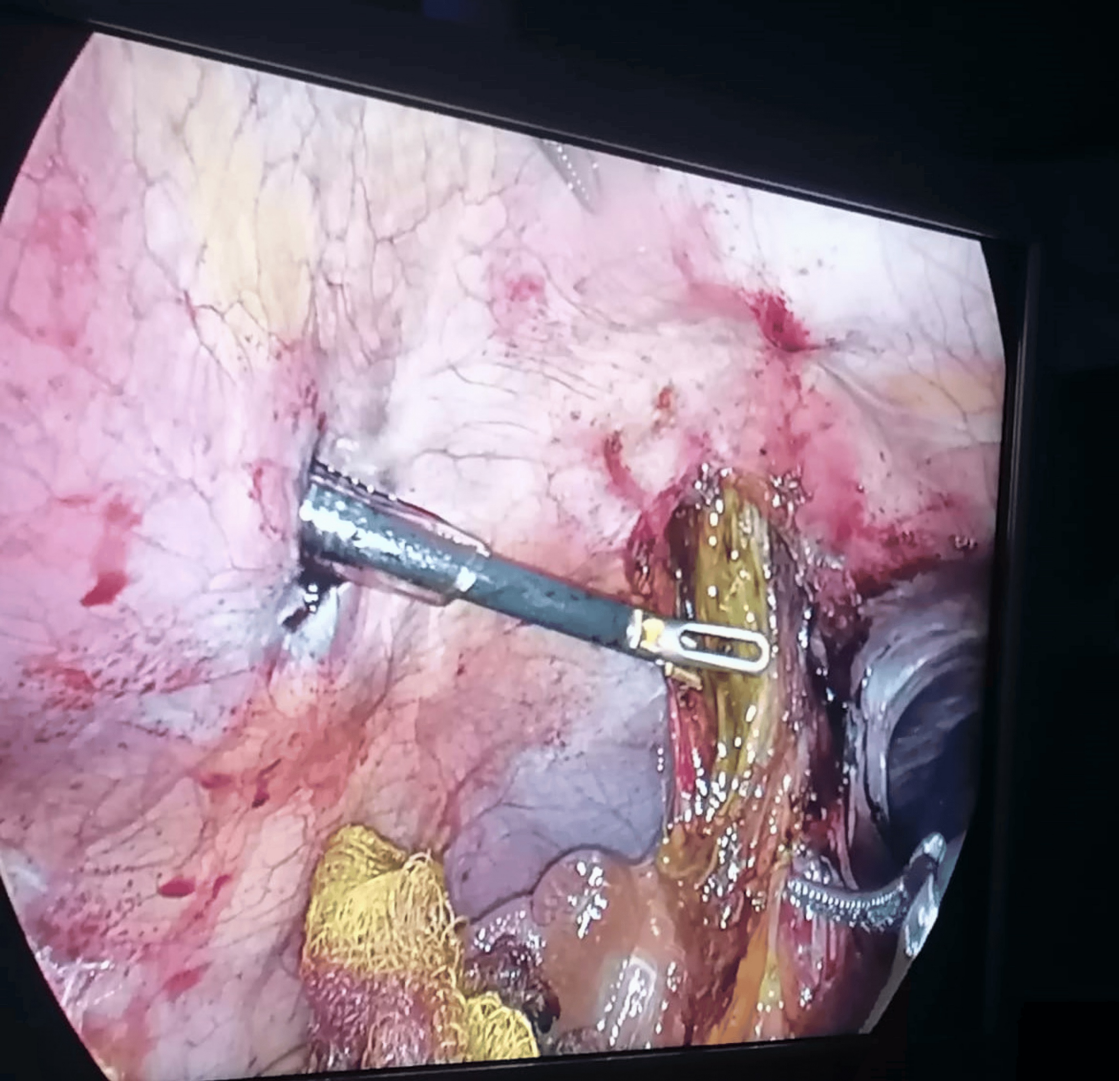
Herniated gallbladder observed during laparoscopic surgery.

The procedure began in a standardized manner following the hospital’s protocol. Laparoscopic ports were placed with direct visualization. During the laparoscopic approach, the gallbladder and great omentum were found herniated in a traumatic diaphragmatic defect (Figure 6). After meticulous dissection, a retrograde partial cholecystectomy was performed due to the presence of extensive scar tissue.

**Figure 7:**
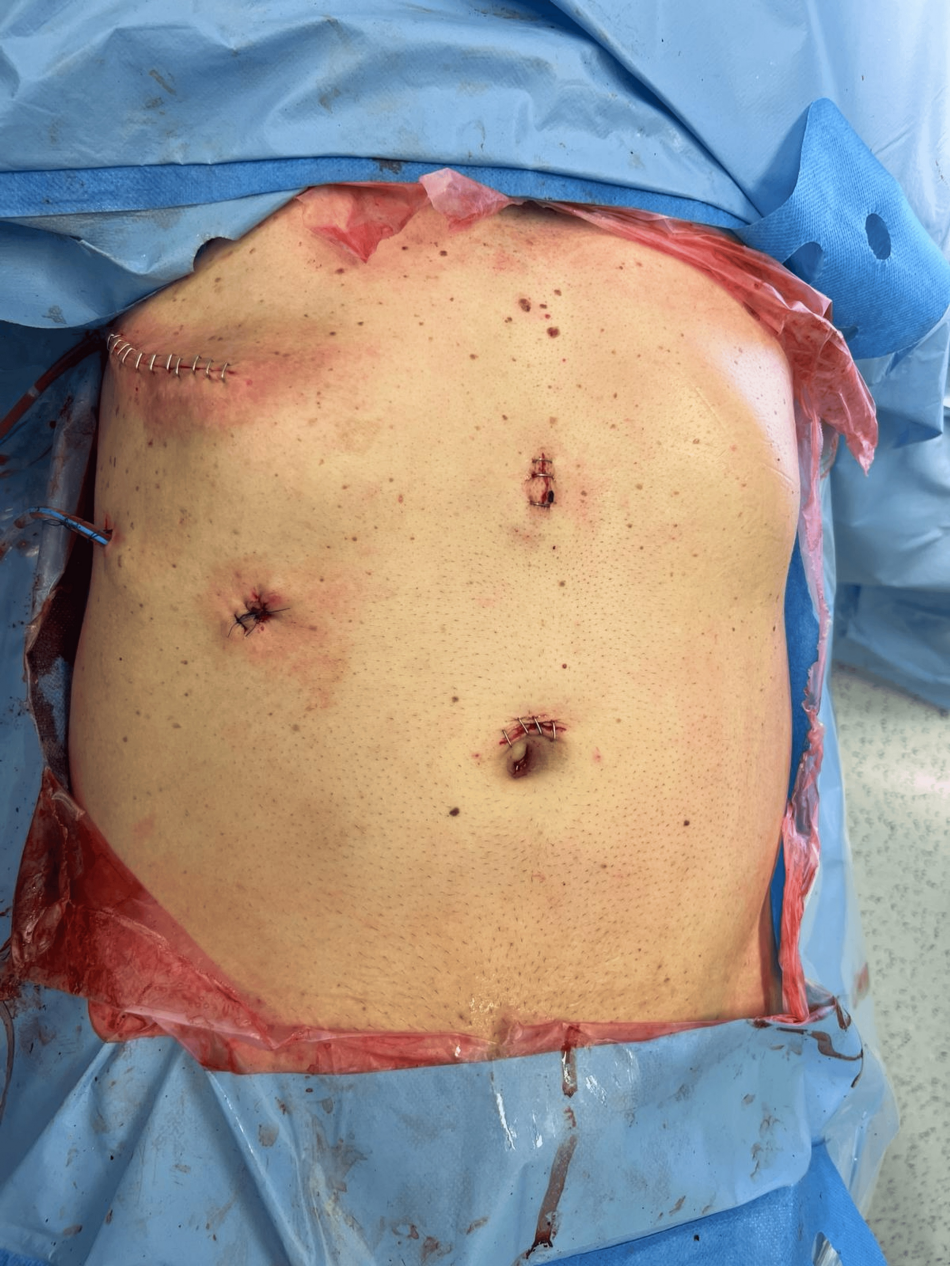
Surgical incisions, immediately after completing the procedures.

Following the laparoscopic procedure, a thoracotomy was performed in the sixth right intercostal space to repair the diaphragmatic tear and intercostal hernia, as this repair could not be accomplished laparoscopically (Figure 7). The patient was discharged on postoperative day three.

**Figure 8:**
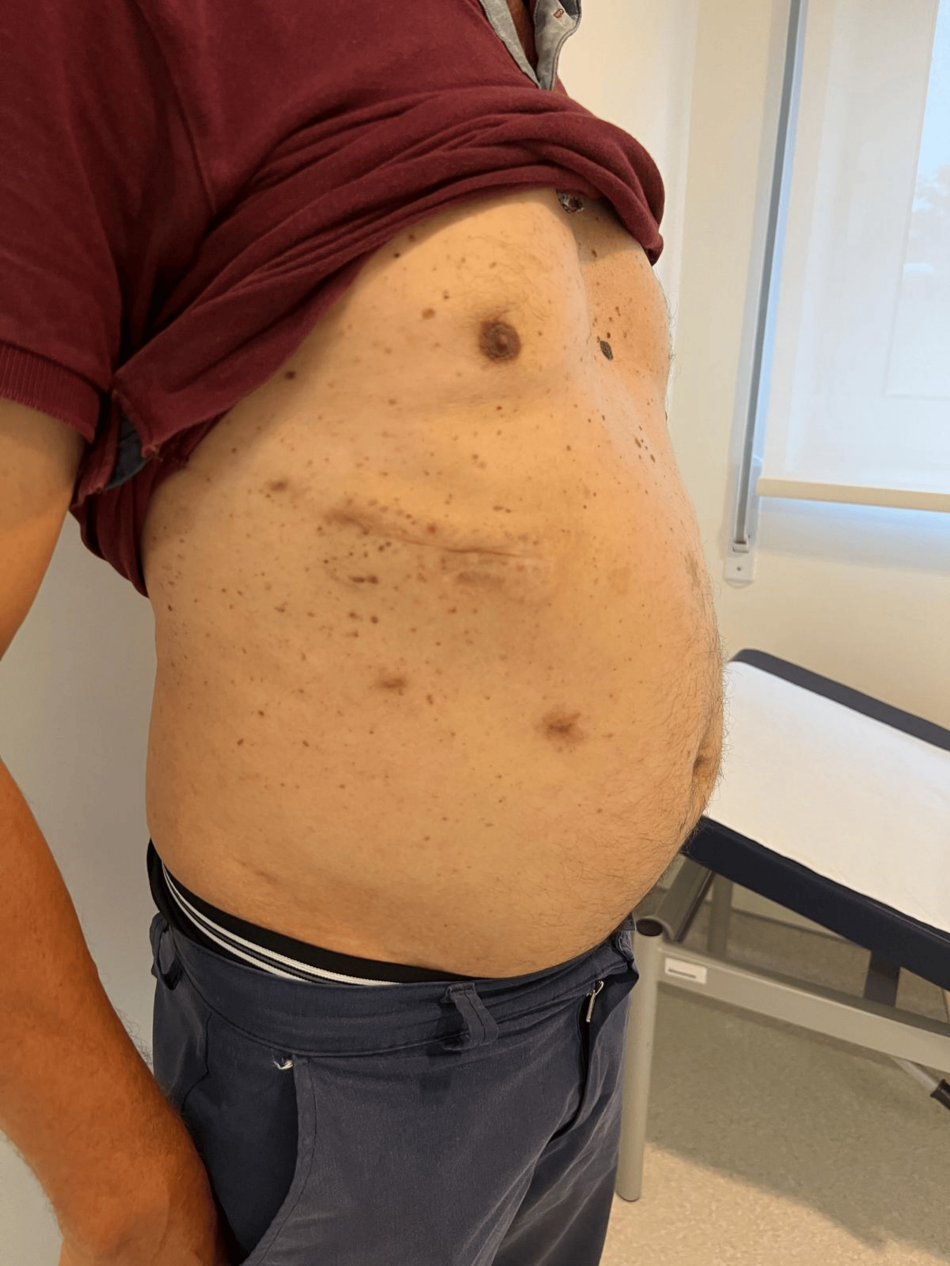
Surgical scars one month after surgery.

In the follow-up appointment, the patient had no complaints and no recurrence of the abdominal wall hernia (Figure 8).

## Discussion

The management of hepatic trauma remains a complex clinical challenge, but it has evolved significantly over the past 25 years. Advances in diagnostic imaging, critical care, and interventional radiology have made non-surgical treatment the preferred approach in most cases of blunt liver injury [7], particularly for hemodynamically stable patients. While management strategies for liver trauma have improved, similar progress has not been seen with gallbladder trauma, largely due to its rarity and the lack of substantial case data.

Most hepatic injuries are low-grade and are managed nonoperatively if the patient is hemodynamically stable [8]. Grade III or higher grade liver injuries often require a combination of surgical and angiographic procedures, but nonoperative treatment might be considered if the patient remains hemodynamically stable and has no other indications for abdominal surgery [9], regardless of the injury grade [10].

When considering a patient for non-operative management, careful selection is critical, and resources for potential intervention must be readily available, including access to interventional radiologists, an intensive care unit, a blood bank, an operating room, and an experienced surgical team [8,11].

In this case, non-operative management was chosen primarily due to the presence of grade III hepatic laceration in a hemodynamically stable patient who showed no signs of complications, such as bile duct injury or infection, during his hospital stay. Elective laparoscopic surgery was successfully and safely performed after the hepatic laceration and rib fractures had healed.

## Conclusions

This case report, to our knowledge, is the first described case of gallbladder herniation following blunt trauma. It was challenging due to the scarce literature concerning the management of gallbladder injury following blunt trauma. This case demonstrates the possibility of unique injury patterns in high-energy trauma and highlights the importance of serial imaging and multidisciplinary management in cases involving complex thoracoabdominal trauma.

Non-operative management was successful due to the patient's hemodynamic stability, self-limited hemorrhage, and absence of complications such as bile duct injury or infection. The patient was ultimately considered for surgery only after discharge, with elective surgery safely performed once healing had progressed.

This case demonstrates the feasibility of conservative management followed by elective intervention for stable patients with traumatic gallbladder herniation, contributing valuable insights to the limited body of knowledge on this condition.
